# Longitudinal Bone Density During TSH Suppression in Differentiated Thyroid Cancer: A Paired PET/CT Analysis

**DOI:** 10.3390/cancers17213462

**Published:** 2025-10-28

**Authors:** Holger Einspieler, Hannah Klimpfinger, Song Xue, Aleksandar Debeljkovic, Bettina Reiterits, Bengt Hennig, Marcus Hacker, Georgios Karanikas

**Affiliations:** Department of Biomedical Imaging and Image-Guided Therapy, Division of Nuclear Medicine, Medical University of Vienna, 1090 Vienna, Austria

**Keywords:** thyroid, PET/CT, TSH suppression, differentiated thyroid cancer, Hounsfield units, bone density

## Abstract

**Simple Summary:**

Many patients treated for differentiated thyroid cancer receive long-term levothyroxine therapy to suppress thyroid-stimulating hormone (TSH) and lower the risk of recurrence. This hormone state can affect several organs, including the skeleton. We examined whether bone density in the lumbar spine declines during suppression. Using routinely performed CT scans, we measured Hounsfield units in each lumbar vertebra at two timepoints with a mean interval of four years. Bone density decreased significantly over time in the entire cohort. In sex-stratified analyses, women showed significant declines at several vertebral levels, whereas men showed a smaller, non-significant downward trend. Age, rather than sex alone, was the stronger baseline determinant of bone density. These findings support regular bone-health monitoring during long-term levothyroxine-induced TSH suppression.

**Abstract:**

**Background**: While TSH suppression is essential in patients with differentiated thyroid cancer (DTC) to reduce the risk of recurrence, it has also been linked to side effects, particularly a reduction in bone mineral density that may contribute to osteoporosis. However, previous studies investigating this association have yielded inconsistent results. This study aimed to evaluate bone density using Hounsfield units from PET/CT scans in a longitudinal analysis including both sexes. **Methods**: Patients with DTC under continuous TSH suppression who underwent two PET/CT scans were included. Hounsfield units were measured for each lumbar vertebra (L1–L5) in the CT by placing an elliptical region of interest (ROI) in the center of the vertebra, avoiding hyperdense edges. Laboratory parameters were also collected. **Results**: A total of 50 patients were included in the study (25 male, 25 female), with a mean age of 57.2 (±15.3) years at the time of the first scan. The mean duration of TSH suppression before the first scan was 3.7 ± 3.9 years, and the mean interval between both scans was 4.4 ± 4.0 years. At the follow-up scan, bone density was significantly lower compared with baseline for all lumbar vertebrae (L1–L5 combined and individually) (all *p* < 0.05). Subgroup analysis revealed a significant decline in women at L1, L2, L4, and L5 and for overall lumbar bone density, while men showed nonsignificant trends. **Conclusions**: Our study suggests a sustained reduction in vertebral bone density during TSH suppression. The results support routine monitoring in both sexes, risk stratification by age and duration of suppression, and, when oncologically appropriate, consideration of lower suppression intensity or initiation of bone-protective therapy in high-risk patients.

## 1. Introduction

Thyroid cancer is the most frequent malignancy of the endocrine system worldwide [1]. The age-standardized incidence is approximately 4.2 per 100,000 in men and 13.8 per 100,000 in women [2]. Over the past four decades its incidence has grown substantially, a trend largely attributable to increased detection from improved imaging techniques and the use of fine-needle aspiration biopsy [3].

Differentiated thyroid cancer (DTC), consisting of papillary (PTC) and follicular (FTC) thyroid cancer subtypes, represents over 90% of thyroid malignancies worldwide [4]. Due to the existence of effective treatment options, this type of cancer has, in general, a favorable prognosis, with a 10 years overall survival rate of 85–98% [5,6]. Standard treatment typically involves total thyroidectomy followed by radioiodine ablation. Postoperatively, patients require lifelong thyroid supplemental hormones [7]. To minimize the risk of recurrence, thyroid-stimulating hormone (TSH) levels are actively suppressed, as TSH can stimulate leftover thyroid cells [8,9]. Biologically, the approach is plausible: DTC cells express TSH receptors; ligand binding activates mitogenic signaling and increases thyroid specific proteins—including thyroglobulin and the sodium-iodide symporter—thereby supporting tumor proliferation [10]. However, while TSH is widely considered a growth stimulus for DTC cells, the foundation for this rationale comes mainly from in vitro studies, with comparatively limited confirmation in clinical settings [11].

TSH suppression has been associated with reduced bone mineral density (BMD), a key factor in the development of osteoporosis. Osteoporosis is defined by reduced BMD and deterioration of bone microarchitecture, leading to an increased likelihood of fragility fractures. The clinical and societal burden is considerable [12]. Vertebral fractures are frequently missed yet contribute to chronic pain, kyphosis and reduced quality of life [13]. Hip fractures are associated with substantial short- and long-term mortality [14]. While low BMD is a central driver of risk, fracture susceptibility is multifactorial and accumulates with age. Established risk factors are advanced age, female sex, prior trauma, history of fracture in a parent, low body mass index, prolonged glucocorticoid therapy, smoking, excess alcohol intake, physical inactivity, and inadequate calcium or vitamin D intake. Several chronic conditions further elevate risk, including rheumatoid arthritis, chronic kidney and liver disease, and chronic inflammatory disorders of the gastrointestinal tract [15,16].

As BMD reflects the balance of bone remodeling, in which osteoclast-mediated resorption is coupled to osteoblast-driven formation and coordinated by osteocystes, hormonal influences on this cycle are relevant. Thyroid hormones including thyroxine (T4) and triiodothyronine (T3) accelerate remodeling: intracellular T3, acting through thyroid hormone receptor- α in osteoblast-lineage cells, promotes differentiation, matrix synthesis, and mineralization. T3 has also been reported to facilitate osteoclastogenesis. TSH signaling is likewise implicated in skeletal regulation: TSH receptors are present on osteoblasts and osteoclasts. Experimental data on osteoblasts are inconsistent, but literature indicates that TSH receptor activation can inhibit osteoclastogenesis and osteoclast activity, potentially by upregulating osteoprotegerin and suppressing tumor necrosis factor α transcription [17]. Given these biologic effects, reliable BMD assessment is critical. While dual x-ray absorptiometry (DXA) is the gold standard for BMD assessment, Hounsfield units (HU) from CT scans have been shown to correlate strongly with DXA and provide an alternative method for evaluating bone health [18,19,20]. Mean HU can be easily extracted from a region of interest (ROI) in CT scans, which were already obtained for other indications, without incurring additional costs or radiation exposure [21].

Furthermore, long-term TSH suppression induces a state of iatrogenic subclinical hyperthyroidism with consequences across multiple other organ systems. Cardiovascular effects are among the best described: tachycardia and atrial fibrillation occur more frequently, and chronic exposure has also been linked to increased left-ventricular mass with subtle diastolic impairment [22,23]. Vascular changes such as arterial stiffness and arterial inflammation have also been reported, potentially amplifying baseline cardiovascular risk [24,25,26]. Beyond cardiovascular effects, patients may experience neuropsychiatric symptoms—including anxiety, sleep disturbance and depression—which can adversely affect daily functioning [27]. The musculoskeletal system, as already described, is likewise sensitive to thyroid-hormone excess, particularly the trabecular-rich vertebrae, where accelerated remodeling may lower bone mass over time [17].

However, evidence on the impact of TSH suppression on bone loss and osteoporosis is inconsistent—with some studies reporting significant reductions in bone density while others finding no effect [28,29,30,31,32]—possibly reflecting small samples, heterogeneous cohorts, and predominantly cross-sectional designs; in the few existing longitudinal observations, follow-up is often short. To clarify this issue, we conducted a longitudinal study in patients under continuous TSH suppression, using a within-subject paired design (two scan timepoints) to reduce between-patient confounding and provide a direct estimate of bone density change. This study partially builds on our previous work [33] but markedly expands the longitudinal cohort of patients under TSH suppression, includes a younger mean age than the prior cohort, and provides a more robust analysis with a larger sample size. Given also the longer TSH suppression interval between both timepoints in this cohort, our findings provide critical information for clinicians regarding the progressive risk of bone loss over time—an issue of practical relevance for long-term monitoring.

## 2. Methods

### 2.1. Patients

In this retrospective longitudinal analysis, we included patients treated for DTC in the Division of Nuclear Medicine at the Vienna General Hospital from 1 January 2016–1 May 2025 who were under continuous TSH suppression and had at least two PET/CT scans, with at least one scan within this time frame and the other permitted outside to maximize the inter-scan interval. The study was approved by the Ethics Committee of the Medical University of Vienna (EK: 1640/2023), and written informed consent was waived for data collection and analysis due to its retrospective design.

**Figure 1:**
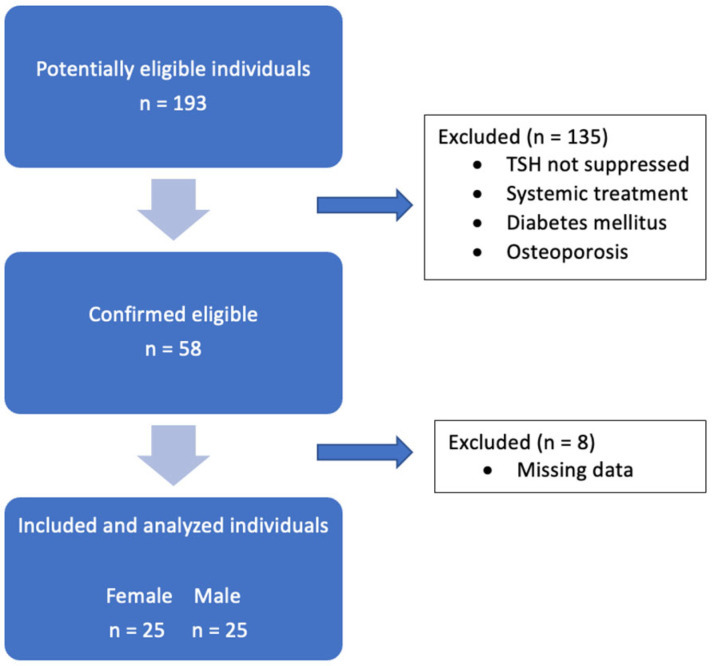
Flowchart summarizing the patient selection process.

Men and women in this study all received primary treatment including total thyroidectomy and radioiodine ablation due to DTC prior to the initiation of TSH suppression. Patients were required to be at least 18 years old, to be at least 6 months under TSH suppression prior to the first PET/CT scan (<0.40 mU/L), and to be under continuous TSH suppression at least until the second PET/CT scan. The exclusion criteria included known diabetes mellitus types 1 and 2, calcium metabolism disorders, severe vitamin D deficiency, osteoporosis, bone neoplasms, routine use of cortisone medications and systemic treatments, including tyrosine kinase inhibitors, bisphosphonates, and chemotherapies. The study flowchart is shown in Figure 1. In brief, 193 potentially eligible individuals were screened. 135 patients were excluded for predefined reasons, as described above. 58 participants fulfilled the initial criteria. Of these, 8 patients were excluded because of missing imaging or clinical data, leaving a final cohort of 50 patients for the longitudinal analyses.

### 2.2. PET/CT Examinations

All whole-body [^18^F]FDG PET/CT scans were performed from the skull base to the thigh using either a Siemens Biograph TruePoint TrueView or Siemens Biograph 128 Vision Quadra Edge PET/CT scanner (Siemens Healthineers, Erlangen, Germany). For the TruePoint TrueView, CT scans used the following parameters: 120 kV, 200–220 mAs, and 5 mm slice thickness. For the Biograph 128 Vision Quadra Edge, CT scans were performed with 120–140 kV, 30–40 mAs, and 2 mm slice thickness.

### 2.3. Image Analysis and Data Collection

The PET/CT images were evaluated using Agfa HealthCare’s IMPAX EE software (DeepUnity Diagnost Version 1.1.0.1, v20210819_0818) by a single reader. A ROI was placed in the lumbar vertebrae (L1 to L5) on the axial section plane of the whole-body bone sequence, aligned parallel to the respective end plates. The largest feasible elliptical ROI was placed while excluding the hyperdense cortical edges of the vertebral bodies and heterogeneous regions, such as the posterior venous plexus and bone islands. The average HU value within the ROI was extracted, which referred to bone density (HU). The measurement of HU values followed the methodology outlined in previously published studies [33,34,35]. Lumbar vertebrae with fractures or tumor involvement were excluded from the analysis.

### 2.4. Statistical Analysis

IBM SPSS Statistics Version 30 was used for the statistical analysis. Descriptive variables were presented as absolute numbers (*n*) or as means with standard deviations (±SD). A paired t-test was conducted to assess the longitudinal changes within the entire cohort (within-subject comparison of baseline versus follow-up). To examine the relationship between variables such as age, duration of TSH suppression, and bone density, either Pearson’s correlation (for approximately normal distribution) or Spearman’s rank correlation (otherwise) was applied, ensuring appropriate handling of distributional differences. Normality of continuous variables was evaluated using the Shapiro–Wilk test. The Mann–Whitney U test was employed to compare the mean changes in bone density between female and male patients (independent groups; non-normal distributions). A two-sided *p*-value of <0.05 was considered statistically significant. Change in bone density (ΔHU) was defined as follow-up minus baseline (mean HU L1–L5 2. PET/CT minus mean HU L1–L5 1. PET/CT); negative values indicate a decrease. Boxplots and scatterplots were used for illustration. Pairwise deletion was applied for missing data, excluding only cases with missing values for specific variables. Two-way analysis of variance (ANOVA) was used to assess the effects of age and sex on baseline and longitudinal bone density (testing main effects and their interaction).

## 3. Results

### 3.1. Patients

In total, 50 patients with a mean duration of 3.7 (±3.9) years of TSH suppression before the first PET/CT scan were included in this study. The cohort comprised 25 women and 25 men. BMI did not differ between baseline and follow-up (*p* > 0.05). Thyroid-related hormones (TSH, fT3, fT4) were stable across timepoints, and serum calcium likewise showed no significant change (*p* > 0.05). Demographic and clinical parameters are demonstrated in Table 1.

### 3.2. Bone Density Comparison Between Baseline and Follow-Up

**Figure 2:**
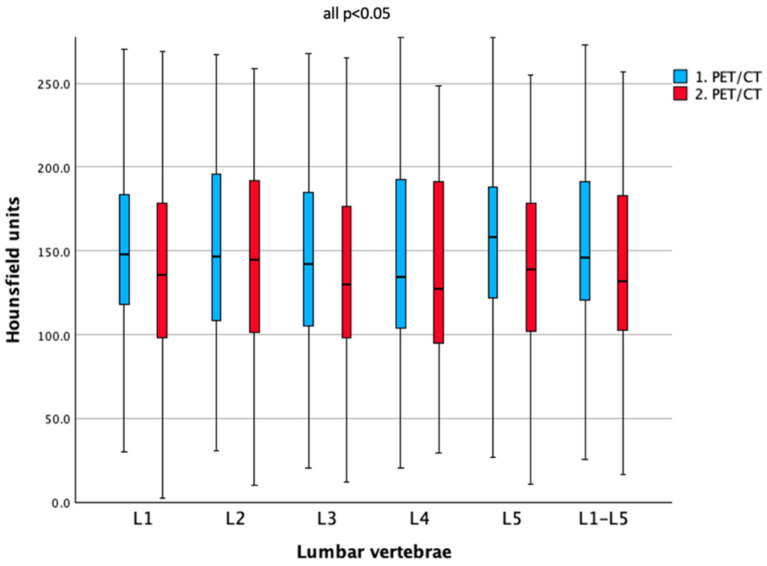
Boxplots of bone density, measured by Hounsfield units, for all lumbar vertebrae across both PET/CT scans under TSH suppression.

Statistically significantly lower bone density (HU) was observed at the second scan compared with the initial scan for L1 (ΔHU = −9.3, 95% CI −17.7 to −0.9), L2 (ΔHU = −9.3, 95% CI −16.9 to −1.7), L3 (ΔHU = −8.4, 95% CI −16.7 to −0.1), L4 (ΔHU = −9.0, 95% CI −16.8 to −1.1), L5 (ΔHU = −19.6, 95% CI −28.6 to −10.5), and the combined lumbar values (ΔHU = −9.8, 95% CI −16.2 to −3.3) (all *p* < 0.05), as can be seen in Table 2 and Figure 2. The mean duration between both scans was 4.4 (±4.0) years.

### 3.3. Correlation Between ΔHU and Years Between PET/CT Scans

**Figure 3:**
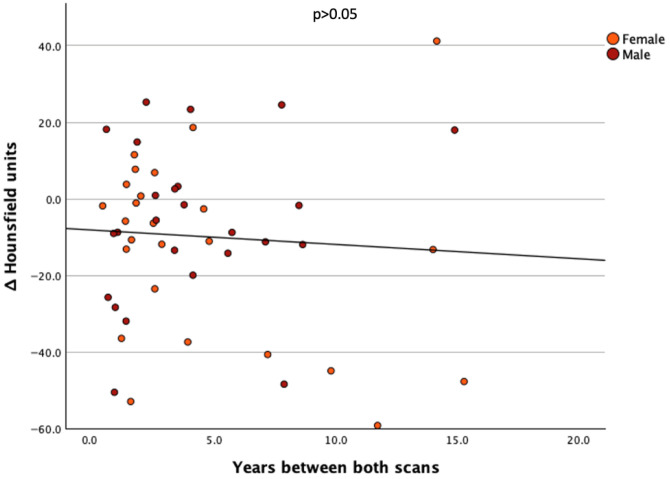
Scatter plot of ΔHounsfield units in all patients under TSH suppression versus the time interval between scans.

Only a very weak negative correlation was observed between ΔHU and the time interval between both scans (Figure 3) (r = −0.066), which did not reach statistical significance (*p* > 0.05).

### 3.4. Sex Differences in Bone Density Outcomes

For evaluating sex-specific outcomes, the study cohort was divided into female and male subgroups.

Firstly, age showed a statistically significant correlation with bone density in both groups, with a strong negative correlation in females (r = −0.753) and a moderate negative correlation in males (r = −0.532).

**Figure 4:**
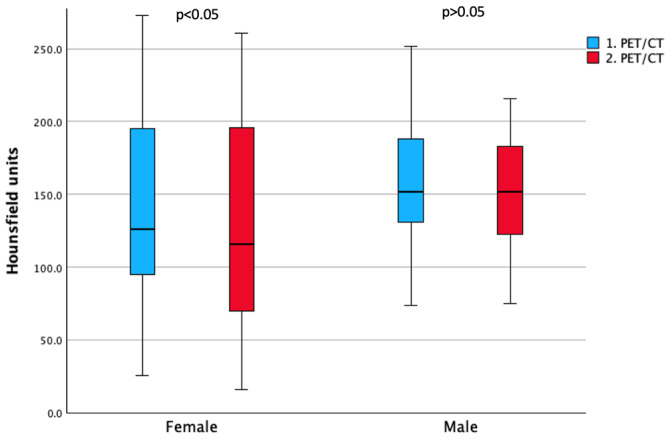
Boxplots of mean L1–L5 bone density (Hounsfield units) in women and men across PET/CT scans.

Secondly, as can be seen in Table 3 and Figure 4, a statistically significant reduction in bone density (HU) was observed only in females at L1, L2, L4, and L5 and for the combined lumbar vertebrae (L1–L5), whereas in males no measurement reached statistical significance.

**Figure 5:**
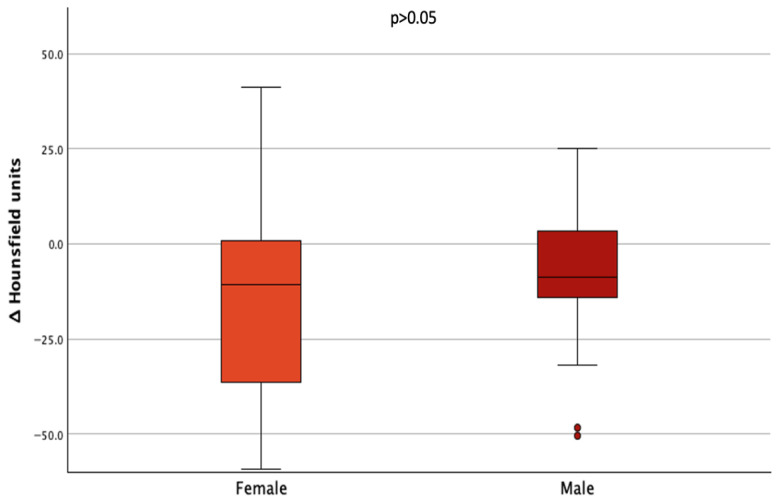
Boxplots of mean changes in ΔHounsfield units of lumbar vertebrae in women and men.

When comparing ΔHU of male to female patients, no statistically significant difference was found (*p* = 0.399) (Figure 5).

### 3.5. Impact of Age and Sex on Baseline and Longitudinal Bone Density (Two-Way ANOVA)

At baseline, two-way ANOVA showed a significant effect of age (>50 years old) and a significant age × sex interaction on bone density (both *p* < 0.05), while sex alone was not significant. In the longitudinal analysis (ΔHU), two-way ANOVA revealed no significant main effects of sex or age group and no significant sex × age group interaction.

## 4. Discussion

The impact of TSH suppression on bone loss and osteoporosis remains controversial, with some studies reporting significant reductions in bone density, while others find no effect [30,32,36,37,38,39].

In our study, the entire cohort showed a statistically significant longitudinal reduction in bone density, measured by HU, across all lumbar vertebrae after a mean of 4 years of TSH suppression, irrespective of sex. Unlike one previous mixed-gender case–control study that found no relevant effect in 65 patients [28], our results are consistent with other reports demonstrating a decline in bone density under TSH suppression in cohorts including both men and women [40,41]. This is further supported by a meta-analysis from Blum et al., which found that subclinical hyperthyroidism is associated with an increased risk of hip and other fractures [42].

Interestingly, when stratifying by sex, we observed a significant decrease in bone density in several lumbar vertebrae among women, whereas in men only a non-significant trend toward lower values was seen. However, when directly comparing ΔHU between sexes, no statistically significant difference emerged, suggesting that the relative impact of TSH suppression on bone loss may be comparable across sexes, although women appear more vulnerable given their lower baseline bone density and the significant within-group decline.

It is also well known that in women, the most pronounced decline usually occurs after menopause, when the loss of estrogen strongly accelerates bone resorption [43,44].

Finkelstein et al. examined 1902 women annually and showed that lumbar spine and hip BMD remain relatively stable in pre- or early perimenopause, but decline markedly from late perimenopause into early postmenopause; body weight further modulated loss rates [44]. Independently of menopausal transition, Guo et al. reported in postmenopausal women on levothyroxine that suppressed TSH is associated with higher bone turnover and lower BMD, and that reducing T4 dose in suppressed patients decreases turnover and increases BMD [29]. In addition, a randomized trial in patients with PTC found that women assigned to TSH-suppressive therapy experienced significant lumbar-spine T-score declines from year 1, whereas the non-suppressed group showed no significant decrease until year 5; the early decline was evident in women ≥ 50 years. By year 5, even non-suppressed patients had significant BMD loss, which the authors assumed reflected natural age-related decline, especially in postmenopausal women [45].

In our cohort, detailed data on menopausal status were unavailable due to the retrospective design, representing a study limitation; however, age appeared to be a stronger determinant than sex: at baseline, we found that increasing age was strongly negatively correlated with lower bone density in women and moderately negatively correlated in men. Furthermore, two-way ANOVA also revealed a significant effect of age and a significant age × sex interaction on bone density, while sex alone was not significant. Longitudinally, as already mentioned, there was a significant decline across multiple lumbar vertebrae in women and a nonsignificant downward trend in men, suggesting that TSH suppression may adversely affect bone in both sexes, with age—and possibly menopausal status—amplifying the effect in women. Unfortunately, we lacked data on menopausal status in this study, which may introduce residual confounding; nevertheless, taken together with prior evidence, these findings support a combined influence of menopause and TSH suppression on bone density. These results support the assumption that especially women are at higher risk of developing low bone density and, consequently, osteoporosis under long-term TSH suppression. Future studies should include detailed menopausal staging to quantify this interaction more precisely.

Notably, in contrast to another longitudinal study [31], we observed that bone density continued to decline even after several years of continuous TSH suppression, underscoring the importance of monitoring bone health over time. In line with this, and in contrast to our previous study with a smaller sub cohort and a shorter imaging interval [33], the present analysis, with a much larger sample size and longer TSH-suppressive treatment duration, reveals significant longitudinal reductions in bone density. Unlike prior cross-sectional or short-term reports, we quantified within-patient longitudinal change in lumbar vertebral density over a mean of 4 years under ongoing TSH suppression. By leveraging HU from routine oncologic CT, we applied a pragmatic approach to skeletal monitoring without additional scans. Because TSH suppression has multisystem effects, this longitudinal perspective is clinically relevant. Cardiovascular changes are well described, including tachycardia and atrial fibrillation [46], and neuropsychatric symptoms such as depressed mood and anxiety have been reported, with potential impacts on daily functioning and adherence to care [27]. From a skeletal perspective, low bone density increases the risk of osteoporosis and injury, particularly in older adults. In those receiving prolonged TSH suppression, accelerated bone turnover may preferentially deplete trabecular bone at the spine, raising the likelihood of vertebral deformities and subsequent fragility fractures [17,47]. Taken together, these potential adverse effects argue for careful, individualized decisions about the need, intensity and duration of TSH suppression based on oncologic risk. When TSH suppression is indicated, routine surveillance of bone health and other potential side effects should be integrated into follow-up—for example baseline and periodic HU/DXA measurements, alongside lifestyle optimization (adequate calcium and vitamin D). Our findings suggest that the intensity and duration of TSH suppression should be individualized to recurrence and skeletal risk, balancing oncologic benefit against skeletal complications, and that anti-osteoporotic prophylaxis may be considered during therapy. These pragmatic approaches could help balance the benefits of suppression with its potential harm.

This study has several limitations. First, its retrospective design and relatively small sample size may limit the generalizability of the findings. Second, bone density was assessed using CT rather than DXA, which is considered the gold standard for evaluating BMD. In addition, the use of two PET/CT scanners may have introduced minor inter-scanner variability in HU measurements. Third, serum 25-hydroxyvitamin D levels were unavailable, which may have affected bone density.

## 5. Conclusions

To sum up, we observed a statistically significant reduction in lumbar bone density at the second imaging timepoint after a mean of four years of TSH suppression, indicating a clinically relevant impact of TSH long-term suppression on bone health. In sex-stratified analyses, women showed significant within-group declines across multiple lumbar vertebrae, while men exhibited a smaller, non-significant downward trend. Given that the between-sex difference in change was not significant, this pattern suggest that TSH suppression contributes to bone loss at the population level. Clinically, these findings support routine bone-health surveillance during prolonged TSH suppression in both sexes, including baseline and periodic bone density assessments, with heightened vigilance in older patients. Management should include optimization of modifiable factors such as adequate calcium and vitamin D intake, and, when oncologically appropriate, consideration of lower TSH suppression intensity or bone-protective therapy in higher-risk individuals. Future studies should prospectively evaluate bone density outcomes in both sexes under TSH suppression, explicitly modeling age and incorporating menopausal status, a potential confounder, to clarify sex-related effects on bone outcomes.

## Figures and Tables

**Table 1:** Demographic and clinical characteristics of the study cohort at both PET/CT scan timepoints.

Parameters	1. PET/CT	2. PET/CT	*p*-Value
Age in years (mean ± SD)	57.2 (±15.3)	61.5 (±15.2)	<0.001
Sex:			1.000
male	25	25	
female	25	25	
BMI (mean ± SD)	27.3 (±4.6)	26.9 (±4.9)	0.116
Blood parameters (mean ± SD):			
TSH (µU/mL)	0.08 (±0.1)	0.09 (±0.2)	0.421
fT3 (pg/mL)	3.2 (±0.6)	3.1 (±0.4)	0.089
fT4 (ng/dL)	1.81 (±0.4)	1.72 (±0.3)	0.256
Calcium (mmol/L)	2.29 (±0.11)	2.25 (±0.15)	0.093

SD = standard deviation; BMI = body mass index; fT3 = free triiodothyronine; fT4 = free thyroxine; TSH = thyroid-stimulating hormone.

**Table 2:** Average bone density in Hounsfield units (HU) of all lumbar vertebrae across both PET/CT scans in all patients under TSH suppression.

Lumbar Vertebrae	Bone Density (HU ^$^)—1. PET/CT	Bone Density (HU ^$^)—2. PET/CT	*p*-Value
L1	149.1 ± 53.1	139.8 ± 59.5	0.031 *
L2	149.5 ± 53.8	140.1 ± 57.9	0.017 *
L3	144.4 ± 55.3	136.0 ± 56.4	0.049 *
L4	145.5 ± 59.8	136.5 ± 59.8	0.026 *
L5	159.9 ± 61.2	140.3 ± 58.1	<0.001 *
Mean L1–L5	150.4 ± 53.9	140.6 ± 56.4	0.004 *

L = lumbar vertebra; HU = Hounsfield units; ^$^ = (mean ± standard deviation); * = statistically significant.

**Table 3:** Average bone density in Hounsfield units of lumbar vertebrae across both PET/CT scans in men and women under TSH suppression.

	Female		Male	
Lumbar Vertebrae	Bone Density (HU ^$^)—1. PET/CT	Bone Density (HU ^$^)—2. PET/CT	Bone Density (HU ^$^)—1. PET/CT	Bone Density (HU ^$^)—2. PET/CT
L1	142.4 ± 62.9 *	128.3 ± 67.7 *	155.5 ± 42.0	150.9 ± 49.2
L2	137.8 ± 63.0 *	126.3 ± 69.7 *	161.1 ± 40.6	153.9 ± 39.8
L3	136.7 ± 65.1	126.5 ± 68.6	152.1 ± 43.5	145.5 ± 40.2
L4	139.4 ± 72.0 *	127.4 ± 71.7 *	151.6 ± 45.2	145.7 ± 44.8
L5	151.6 ± 66.8 *	123.5 ± 66.5 *	168.1 ± 55.3	157.1 ± 43.4
Mean L1–L5	142.6 ± 64.1 *	129.4 ± 68.9 *	158.2 ± 41.3	151.8 ± 38.5

L = lumbar vertebra; HU = Hounsfield units; ^$^ = (mean ± standard deviation)*;* * = statistically significant difference between both scans.

## Data Availability

The datasets generated and/or analyzed in this study are available from the corresponding author upon reasonable request.

## Abbreviations

ANOVA, analysis of variance; BMD, bone mineral density; DTC, differentiated thyroid cancer; DXA, dual x-ray absorptiometry; FTC, follicular thyroid cancer; HU, Hounsfield unit; PTC, papillary thyroid cancer; ROI, region of interest; SD, standard deviation; TSH, thyroid-stimulating hormone; T4, thyroxine; T3, triiodothyronine.
